# Supplementary material for: Livelayer: a semi-automatic software program for segmentation of layers and diabetic macular edema in optical coherence tomography images
Source: Sci Rep. 2021 Jul 2;11:13794. doi: 10.1038/s41598-021-92713-y (PMC8253852; doi:10.1038/s41598-021-92713-y)
Supplement: Supplementary file 2 — Supplementary Note. [file 41598_2021_92713_MOESM2_ESM.docx]

**Livelayer: A Semi-Automatic Software Program for Segmentation of Layers and Diabetic Macular Edema in Optical Coherence Tomography Images**

Mansooreh Montazerin^1^, Zahra Sajjadifar^1^, Elias Khalili Pour^2^, Hamid Riazi-Esfahani^2^, Tahereh Mahmoudi^3^, Hossein Rabbani^4^, Hossein Movahedian^5^, Alireza Dehghani^5^, Mohammadreza Akhlaghi^5^, Rahele Kafieh^4*^

^1^Department of Electrical and Computer Engineering, Isfahan University of Technology, Isfahan, Iran

^2^Retina Service, Farabi Eye Hospital, Tehran University of Medical Sciences, Tehran, Iran

^3^Department of Biomedical Systems and Medical Physics, Tehran University of Medical Sciences, Tehran, Iran

^4^Medical Image and Signal Processing Research Center, School of Advanced Technologies in Medicine, Isfahan University of Medical Sciences, Isfahan, Iran

^5^Department of Ophthalmology, Isfahan University of Medical Sciences, Isfahan, Iran

*Corresponding Author

Rahele Kafieh

Medical Image and Signal Processing Research Center, School of Advanced Technologies in Medicine, Isfahan University of Medical Sciences, Isfahan, Iran

[rkafieh@gmail.com](mailto:rkafieh@gmail.com)

**Supplementary Note**

Here, we bring a concise explanation for how the Livelayer algorithm should be applied over the intended image by the user. Explanations regarding other parts of the proposed software are provided in the supplementary video file.

First of all, the user should choose the desired dataset as the software’s input which could be of three various file formats (i.e. .mat, .vol or .octbin) on the left hand side of the “File” tab. In the “Auto Layer Segmentation” tab which embodies the Livelayer method, he should click on the initial pixel of the desired path and move the mouse along that path, waiting for the live-wire to displays the smallest cost path based on the brightness of pixels in the original image. The user should then drag the mouse on the path so as to discover a route that nicely fits that path and pause the live-wire by clicking on, whenever he observes that the route has become inappropriate. He could resume by clicking on the previous pixel beside the last one and this process proceeds until the entire path is acquired. Finally, the attained boundaries are passed through a smoothing stage and are plotted on each B-Scan while their corresponding coordinates are saved in a “.mat” file, altogether forming the software’s main output. The semi-automatic fluid segmentation section requires the same procedures that are clarified for the Livelayer method and outputs all the segmented fluids’ coordinates along with their mask images (a black background image in which the segmented fluids are presented as white objects).
